# Assessing the burden of oral health conditions in the German population: a focus on dental caries, periodontitis, and edentulism

**DOI:** 10.1186/s12903-026-08134-8

**Published:** 2026-03-19

**Authors:** Meyyammai Meyyappan, Laura Krause, Alexander Rommel

**Affiliations:** 1 Institute of Public Health, Charite – Universitätsmedizin Berlin, Freie Universität Berlin and Humboldt-Universität zu Berlin, Institute for Public Health, Chariteplatz 1, Berlin, 10117 Germany; 2 Department of Epidemiology and Health Monitoring, Robert Koch Institute, Gerichtstraße 27, Berlin, 13347 Germany

**Keywords:** Oral health, Dental health, Dental caries, Periodontitis, Edentulism, Burden of oral disease, YLD, Years lived with disability, DMS V, Germany

## Abstract

**Background:**

Oral diseases are a major public health problem globally. In Germany, there is a high utilization of dental services, and oral health has significantly improved over the past decades. However, a more specific assessment of burden exerted by oral health conditions, namely carious disease, periodontitis and edentulism is required. In contrast to the Global Burden of Disease (GBD) study which often relies on multiple sources of data to report its estimates, this study uses a nationally representative dataset to provide a focused and detailed assessment of the oral disease burden in Germany.

**Methods:**

This study analyses nationally representative, cross-sectional oral health data collected by the Institute of German Dentists (IDZ) as part of the Fifth German Oral Health Study (DMS V) study conducted from 2013 to 2014. The data consists of clinical oral health examinations and socio-demographic information for predefined age groups. Prevalence of carious disease, periodontitis, and edentulism was estimated using standardized clinical indices. Disease burden was quantified as Years Lived with Disability (YLDs), applying disability weights and severity distributions from the GBD framework. Estimates were stratified by age and sex, weighted to reflect the German population and compared with corresponding GBD estimates.

**Results:**

The burden of Carious Disease is higher in males than in females. In females aged 35–39 years, the burden of severe carious disease was nearly double that of mild cases, 55.1 YLDs per 100,000 compared to 25.9 YLDs per 100,000, respectively. The prevalence of periodontitis exceeded 97.5% in adults aged 65–69 years, with an approximate burden of 700 YLDs per 100,000 across both sexes. Edentulism affected 47.2% of females aged 85 years and older and had the highest burden of 3162.4 YLDs per 100,000 people.

**Conclusions:**

This study highlights the marked burden exerted by oral diseases specifically periodontitis and edentulism, especially in older age groups. There are notable sex differences across the oral diseases, and nationally representative DMS V data fills the gaps present in modelled data used be the GBD. Future research can focus on assessing burden estimates for oral conditions under treatment to elucidate differences in treatment strategies and public health decisions.

**Supplementary Information:**

The online version contains supplementary material available at 10.1186/s12903-026-08134-8.

## Background

Oral diseases rank among the most prevalent noncommunicable diseases (NCDs) in modern societies and require a substantial due of health care systems [1]. In 2021, the main oral conditions (untreated caries, severe periodontitis, edentulism, and other oral disorders) affected an estimated 3.69 billion people worldwide [2]. Untreated caries of permanent teeth remained the most prevalent condition of the 371 diseases and injuries assessed in the Global Burden of disease (GBD) study in 2021, recording 2.24 billion cases worldwide. Severe periodontitis recorded 1.07 billion cases, while edentulism recorded 353 million cases globally [2].

Europe has displayed a decreasing trend in age-standardized prevalent cases and Disability-adjusted life years (DALY) rates of these oral conditions from 1990 namely, -24.1% percent change in cases of untreated caries in deciduous teeth, -6.84% percent change in cases of untreated caries in permanent teeth, -25.7% percent change in cases of severe periodontitis and -43.6% percent change in cases of edentulism; a stark contrast compared to African and Eastern Mediterranean regions which display an increasing trend [2].

In Germany, epidemiological data concerning the prevalence of oral diseases nevertheless show that both dental caries and periodontal diseases are highly prevalent, a situation similarly shared with European and non-European developed nations [3, 4]. However, there has been a consistent improvement in the oral health of children, with nearly 78.5% girls and 76.7% boys in the age of 12 years that were reported to be caries-free in 2021 [5].

Untreated oral diseases have numerous consequences, mainly functional limitations and greatly affect emotional, physical and mental wellbeing [1, 6, 7]. The burden of oral diseases is often not uniformly distributed across populations [1]. There are different socio-economic gradients and population groups that are disadvantaged which tend to be affected more by this [7, 8]. People who are in the low education and income group, people living with disabilities, the geriatric population, refugees, people in prison or living in remote and rural communities, children and people from minority and/or other socially marginalized groups generally carry a higher burden, similar to other NCDs [1, 6, 8].

Dental care in Germany is predominantly organized within a statutory health insurance system, which covers approximately 88–90% of the population, while the remaining population is insured privately or through special schemes [9]. Dental services are largely delivered in an outpatient setting by self-employed dentists. Krause et al. [10] reports that contract dental care in Germany under statutory health insurance also covers various services in comparison to other countries. The majority of adults in Germany (82.3%) have utilized dental services within one year, which is 20% points higher than the European average utilization (61.1%) [10]. Statutory insurance fully covers regular dental check-ups twice a year, group and individual prophylaxis programs for children and adolescents, and periodontal screening examinations for adults, alongside incentives for regular attendance through bonus schemes that reduce patient co-payments for prosthetic care [9, 10]. This prevention-oriented approach has led to the highest utilization of dental services in the EU and has been associated with substantial improvements in oral health outcomes over recent decades, particularly in younger cohorts [3, 8, 10].

The GBD study is the largest and most comprehensive effort to quantify health loss across places and over time, to improve health systems [11, 12]. From an epidemiological perspective, assessment of the burden of disease in the general population is becoming increasingly important to assist in health policy decision making [11]. DALY measures the total burden of disease comprising mortality (years of life lost due to death, YLL) and morbidity (years lived with disability, YLD). Given that mortality as a direct consequence of caries, periodontitis and edentulism is considered implausible, no deaths due to oral diseases are assumed in the GBD study [13]. In case of oral disorders, YLD is therefore equivalent to DALY– representing a measure used to quantify the burden of health conditions that cause disability in terms of year(s) of “healthy life” lost due to disability [12].

Across countries around the world, the burden of oral conditions has remained largely unchanged and at a high level over the past 30 years [2]. Edentulism, severe periodontitis and lip and oral cavity cancer caused the highest global burden, as demonstrated by their counts of DALYs and age-standardized DALY rates in 2021, namely, 112 (72.3 to 157) per 100 000 population, 80.9 (32.5–165) per 100 000 population and 67.7 (61.3–73.2) per 100 000 population, respectively [2]. These findings highlight the double challenge of controlling the occurrence of new cases of oral conditions and addressing the huge unmet need for oral health care now faced by an unparalleled proportion of the global population [2].

Currently, the GBD study uses a variety of data sources for their estimations in Germany, including population health surveys, health claims insurance data and oral surveys [14] *(Sources listed from GBD 2021 Sources Tool in Appendix A1*). Nevertheless, the estimates from the GBD study often rely on indirect data sources and use modern Bayesian modelling techniques to combine epidemiological data on disease occurrence, disease consequences, and risk factors in order to improve the conclusiveness of the findings [12, 15]. One of the limitations often mentioned is the inconsistency in data availability. In cases of unavailable data, the estimation relies on statistical modelling and use of covariates [2]. This research aims to achieve a more focused and comprehensive assessment by using a single data source, representative of Germany. It will explore the usability of the dataset for the burden of disease analyses and also draw a comparison with the resulting estimates from the GBD study, in order to obtain an alternative picture of the oral health status of Germany in 2014.

Going more beyond the focus of the GBD study, the present analyses also aim to highlight potential health inequalities due to differences in socio-economic status in the population which may contribute to disproportionate burden of oral disease. By doing so, this research intends to improve theoretical and methodological approaches for burden assessment at a national level and further contribute to oral health policy and prevention strategies.

## Methods

### Study design and sample

The Fifth German Oral Health Study (DMS V) is a cross sectional, multi-centre, nationwide representative, socio-epidemiological study conducted by the Institute of German Dentists (IDZ, Institut der Deutschen Zahnärzte) to investigate the oral health status and oral health behaviour of the German resident population in four age groups. This study is registered at the German Health Services Research Data Bank (DMS V registration number VfD_DMSV_13_002152) [3].

The data from the Sixth German Oral Health Study (DMS • 6), which was conducted from 2021 to 2023 [16], cannot yet be used by external scientists for statistical analyses. Therefore, the present analyses are based on the DSM V dataset which contains information on dental health, demographics, and clinical indices.

Ninety study sample points were selected spread across Germany- with 60 study sample points in West Germany and 30 study sample points in East Germany, for reasons of comparison. Multistage stratified random sampling was done and in each age cohort 1,000 subjects (net) were aimed to include into the study [3]. There were 4 data collection teams - each including 1 dentist, 1 interviewer and 1 contact person, along with a backup team – all for whom calibration and inter reliability tests were also performed to ensure validity [3].

The main inclusion criteria were to be residents of Germany, to be within the specified age cohort and provide consent to participating in the study. Individuals under long term hospital or institutional care, that were outside the specified age cohort and those that were unable to consent were excluded from the study. 2,000 people per age cohort were randomly drawn as a gross sample from the records of the local residents’ registration offices in order to achieve a net 1,000 subjects into the study [3]. For older adults, the gross number was 3,000 - due to difficulty in accessibility in order to achieve the necessary net sample size [3]. Participation in the study required written and signed informed consent from the selected participants. For participants aged 12 years, written informed consent was obtained from their parents or legal guardians. In cases where adult participants were under legal guardianship, written consent was obtained from the respective legal guardians. All participants received information as provided by the interviewer about the purpose of the study and a declaration on data protection [3].

The net study sample finally consisted of 4,609 participants, namely 1468 (12-year-olds), 966 young adults (35- to 44-year-olds), 1042 young elderly (65- to 74-year-olds) and 1133 older elderly (75- to 100- year-olds) that were randomly drawn from local residents’ registration offices [3].

### Variables

DMS V has collected a range of dental health and socio-economic variables, which in the following has been used in this research.

Socio-economic status along with sex and age has been utilized. Socio-economic status of individuals is categorized into low-, middle- and high-income group and was based on a model of three variables (school level, occupational position, household income) [3]. The broad age groups were further divided into the following smaller sub-groups, each spanning a 4-year range to ensure better resolution and visualization of discrepancies in dental health and burden across different age ranges:

- 12 years of age
- 35–39 years
- 40–44 years
- 65–69 years
- 70–74 years
- 75–79 years
- 80–84 years
- 85 years and older (as a combined group due to lower frequency of observations)

#### Carious disease of permanent teeth

Given the available age groups, deciduous caries is not taken into consideration for this study. The DMFT Index is a standardized index used to measure the caries status of permanent teeth [17]. It is made up of separate components and has been used in this analysis to compare the burden of dental caries more specifically.

The permanent dentition status of each tooth (crown and root) is recorded as a score from 0 to 9, with 0 to record a sound crown/root with no evidence of treated or untreated clinical caries and 9 to denote a crown/root which cannot be recorded like in cases of severe hypoplasia [17]. The stages of caries that precede cavitation, as well as other conditions similar to the early stages of caries, are excluded because they cannot be reliably identified in most field conditions in which epidemiological surveys are conducted [17].

The DMFT- Index is the sum of the number of Decayed, Missing due to caries, and Filled Teeth in the permanent teeth. The mean number of DMFT is the sum of individual DMFT values divided by the sum of a population [17]. By assessing the findings on tooth level, the DMFT sum score can be calculated (T = teeth). If at least one tooth surface is carious or filled, the whole tooth is classified as a DMF tooth [3]. The DMFT-Index is made up of the following components:

- DT component (D = decayed) stands for tooth or surface destroyed by caries.
- MT component (M = missing) for tooth or surfaces extracted due to destruction by caries.
- FT component (F = filled) for a filled tooth or surface due to caries [3].

In order to determine the carious burden more specifically, an assumption is drawn from the T-Health index [18]. Rather than calculating the number of prevalent cases in terms of population, this converts prevalence into number of prevalent carious teeth, which serves as the basis for calculating the burden of carious teeth as follows:

- The number of decayed teeth per study participant is calculated using the DT-index component of the DMFT index, in line with the above assumption [18]. This measurement is a point prevalence which does not account for filled teeth that could have been decayed earlier in the same year.
- To compensate for this limitation, a mean number of filled teeth is calculated using the FT-index component of the DMFT index assuming filled teeth to carry half of the burden of DT-index. The T-Health index was found to be more strongly associated with perceived oral health when assigning half the weight of a decayed tooth to a filled tooth by keeping the weight of the latter ≤ 0.50(2) [18].
- The number of affected teeth in the population was then calculated by multiplying the number of prevalent case (persons) per age and sex group by (i) the mean number of decayed teeth and (ii) the mean number of filled teeth with the corresponding burden. The total number of affected teeth in 2014 in Germany was obtained by summing up the age and sex specific estimates of the number of decayed and filled teeth in the population.

#### Periodontitis

The CPITN or CPI Index (Community Periodontal Index of Treatment Needs) has been used to measure periodontal health by including ‘Loss of Attachment’ as a measurement and is also part of the Oral Health Surveys of the World Health Organization (WHO) [19–21].

The periodontal status is assessed with a specialized periodontal probe and examines 10 index teeth (representative of the full mouth) for occurrence of gingival bleeding, presence of supra- and subgingival calculus, periodontal pockets with probing depths between 3.5 and 6.0 mm, and clinical attachment loss [20]. This is known as Pocket Probing Depth (PPD) or Pocket Depth (PD) [19, 20]. The DMS V categorizes this into three levels of severity depending on attachment loss i.e. < 4 mm (which is not inflammatory), 4–5 mm (early stages of the inflammatory disease) and > 6 mm (inflammatory disease) [19]. For this analysis, all individuals that were found to have more than 4 mm of attachment loss from the CPI Index are considered as periodontally compromised.

#### Edentulism

Total Tooth loss is a complex, final outcome which has the capability to reflect an individual’s history of dental disease and its treatment by dental services over the years. Edentulism also is a useful epidemiological health marker which is easy to measure [3, 22]. The number of teeth present in the individuals included in the dataset is used to assess the edentulism. The extent is expressed as number of teeth present during time of examination [3].

### Statistical analysis

A weighting factor that is included in the dataset was applied when estimating prevalence to account for the sample’s representativeness of the broader population in 2014 [3]. This adjustment ensured that the final analysis provided more accurate estimates of prevalence, factoring in sampling design and demographic differences between the sample and the.

general population. Before starting statistical analysis, a design loading was conducted to eliminate the disproportional sampling procedure on West/East Germany level [3]. All prevalence estimations were calculated using survey procedures in STATA/SE 17.0, providing both point estimates and confidence intervals. Prevalent cases in terms of persons were calculated by applying prevalence in percent to the official population numbers for 2014 in Germany.

Microsoft Excel was used for YLD calculations and data transformations, including conversions of individual carious cases in terms of persons into tooth-based metrics by applying the T-Health index.

The basic assumptions and concepts to calculate the prevalence and diseases burden is taken from the disease model of the GBD study 2017 [12, 23]. Disease burden is calculated based on number of affected teeth in case of carious and in terms of people in case of periodontitis and edentulism. This is done to better stratify and quantify the extent of the carious burden by analyzing them in the form of affected teeth. YLDs are calculated and reported in total and as rates per 100,000 people.

#### Burden of carious disease of permanent teeth

The GBD case definition for dental caries is “teeth with unmistakable coronal cavity at dentin level, root cavity in cementum that feel soft or leathery to probing, temporary or permanent restorations, or missing teeth extracted due to a caries lesion” [12] The GBD definition of disability associated with symptomatic dental caries is “this person has a toothache, which causes some difficulty eating.” The disability weight associated with this condition is 0.01 (0.005–0.019), as derived from the GBD Disability Weights study [12, 23]. Individuals with active carious experience do not necessarily experience symptomatic tooth pain consistently [12]. GBD further considers the duration and severity of the tooth pain at two levels – mild and severe. Thus, for carious teeth, total YLD is considered as the sum of burden resulting from these two severities for symptomatic carious individuals.

The data transformations described above provide the number of carious prevalent teeth among the German population in 2014, per age group across both sexes. Carious prevalence is therefore expressed in terms of affected teeth rather than individuals. According to the GBD disease model, the adjusted number of carious teeth is divided into distinct severity grades with certain durations based on the findings of extensive literature reviews and meta-analyses, where severities are expressed as a proportion of cases. Duration of carious disease was set on a duration of 1–2 years meaning that by default-duration is 1 year for all caries cases [12]. Based on this, carious teeth were first separated into asymptomatic and symptomatic cases. Symptomatic cases are further divided into mild and severe cases.

Duration of mild cases is assumed to be one year. For duration of severe cases, two phases of pain due to caries were considered. The “initial” phase characterized by periodic pain that is assumed to occur an average of one hour per day and the “terminal” phase as a period of constant symptoms at the end of an episode.

The proportions of asymptomatic, mildly symptomatic, severely symptomatic carious cases and assumed durations of pain, are taken directly from the GBD 2017 oral disorders model [12]. Specifically, severity distributions for permanent caries are derived from Table 6 in the Appendix of the GBD 2017 Oral Disorders Annex [12], which summarizes evidence from published studies on tooth pain due to caries and distinguishes between data-rich and other settings [12].

For permanent teeth, the GBD 2017 model assumes that 39.8% of prevalent carious cases remain asymptomatic, 41.2% experience mild symptoms, and 18.9% experience severe symptoms. Although these severity distributions and duration assumptions are not country-specific, their use ensures consistency with the GBD framework and allows direct comparison between empirically derived national estimates and model-based global burden estimates.

The following calculations were done to transform affected teeth into YLD [12] taking into consideration leap years for improved accuracy.:

First, for 39.8% of the prevalent cases (= share of time in prevalence because one case is considered one year) no burden would be assumed because they are assumed to remain asymptomatic.

In addition, prevalent cases are categorized further into mild and severe cases.

- ➢ 41.2% of the carious cases are assumed to suffer from mild symptoms with occasional pain of 1 h per day. This implies that burden only occurs for YLD = total prevalent cases * 0.412 * 365.24 / 8,765.82 * DW; where 365.24 is average days per year in this case equaling hours under pain per year; since 8,765.82 is hours per year by dividing the hours under pain by 8,765.82 we receive years under pain for mild cases with occasional pain.
- ➢ 18.9% of the carious cases are assumed to suffer from severe symptoms.

(a) 55.2 days under permanent pain leading to a burden of YLD = total prevalent cases *0.189 * 55.2 / 365.24 * DW and (b) suffering the rest of the year under occasional pain leading to a burden of YLD = total prevalent cases*0.189*(365.24–55.2) / 8,765.82 *DW; under (a) by dividing days under pain by 365.24 we receive years under pain and (b) by dividing the hours under pain by 8,765.82 we receive years under pain. Thus, the YLD for severe cases is considered the summation of the YLD for mild and severe cases with YLD for severe cases being the sum of periods under permanent and occasional pain.

**Fig. 1:**
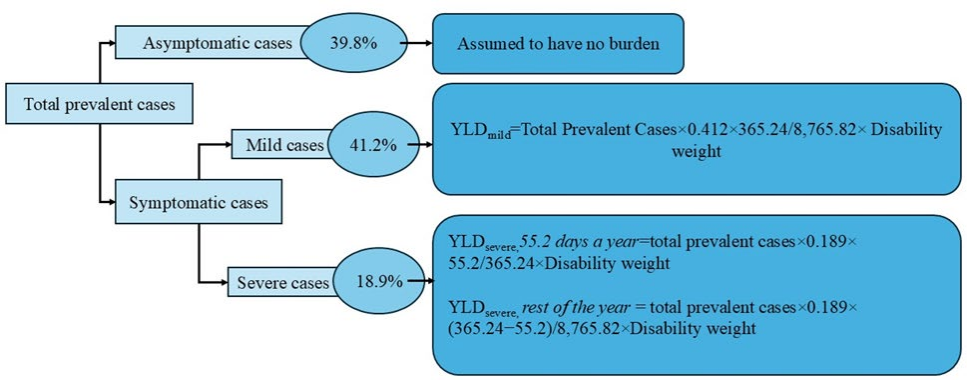
Methodological approach to calculate Years Lived with Disability (YLD) based on severity of carious conditions, adapted from GBD methodology [12]

The methodological approach used to calculate YLD based on the severity of carious conditions is summed up in Fig. 1, adapted from the GBD methodology [12].

#### Periodontitis

The percentage of people suffering from attachment loss is applied to the general population of Germany in 2014 to obtain number of periodontal prevalent cases at the time, by age groups and across both sexes.

The GBD definition of disability associated with symptomatic severe periodontal disease is “bad breath, a bad taste in the mouth, and gums that bleed a little from time to time, but which do not interfere with daily activities” [12]. The GBD disability weight for the condition is 0.007 (0.003–0.014) [12].

The duration of periodontitis is considered to be one year and therefore assumes chronicity. Thus, the YLD is obtained by multiplying prevalent cases with the disability weight of 0.007.

Periodontal YLD = Prevalent cases of periodontitis * 0.007.

#### Edentulism

The case definition of edentulism includes any individual with zero remaining permanent teeth while edentulism of infancy is not included [12].

The assessment of this disease includes quantification of the prevalence of the disease as well as estimation of the major sequelae: asymptomatic toothlessness and symptomatic toothlessness leading to “great difficulty in eating meat, fruits, and vegetables” [12]. The disability weight used for symptomatic toothlessness leading to “great difficulty in eating meats, fruits, and vegetables” is 0.067 (0.045–0.095) as determined by the GBD Disability Survey. All those with severe tooth loss and no access to dentures are considered uniformly to experience this disability [12]. The percentage of edentulous individuals living without dentures is applied to the population in 2014 to calculate number of individuals that would have been suffering from edentulism at the time. The duration is considered to be one year assuming edentulism to be chronic. Thus, the YLD is obtained by multiplying prevalent cases with the disability weight of 0.067.

Edentulism YLD = Prevalent cases of edentulism ∗ 0.067.

The total burden of oral disease is obtained by the summation of all YLDs previously calculated, across both sexes and age groups.

## Results

### Carious disease of permanent teeth

**Fig. 2:**
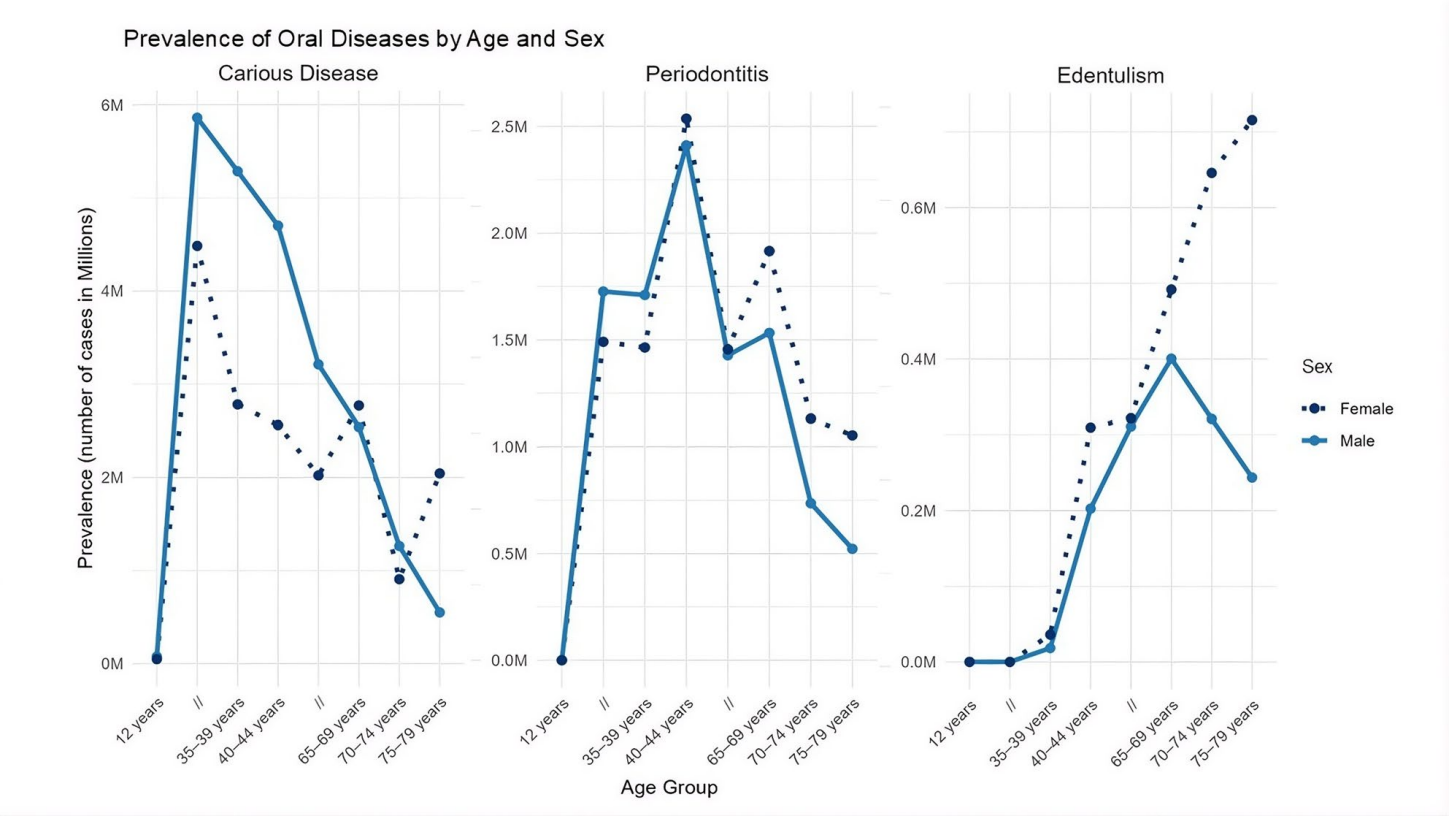
Prevalence (in millions) of oral diseases by age and sex. carious disease is displayed as number of teeth; periodontitis and edentulism are displayed as number of people

Dental caries prevalence, measured by DT Index (decayed teeth), shows an initial age-related increase for both sexes, which gradually plateaus – as illustrated in Fig. 2. Males, aged 65–69 years showed the highest prevalence of 32.0% in comparison to females, where the highest prevalence was observed in 35–39 years of age with about 25.0% (Table 1). Across nearly all age groups, males exhibited a higher prevalence of untreated carious lesions than females.

**Table 1:** Prevalence of carious disease (in %), calculated using decayed tooth (DT) index, with 95% confidence intervals (CI)

Age group	Female	Male
	% (95% CI)	% (95% CI)
12 years	4.2 (2.8–6.2)	7.1 (5.2–9.6)
35–39 years	25.0 (18.8–32.4)	29.3 (22.3–37.3)
40–44 years	15.4 (11.2–20.8)	29.8 (23.6–36.8)
65–69 years	15.5 (11.0-21.5)	32.0 (25.3–39.6)
70–74 years	15.3 (11.0-20.9)	23.2 (17.8–29.6)
75–79 years	20.8 (15.7–27.0)	22.9 (17.9–28.8)
80–84 years	12.4 (7.6–19.4)	24.3 (16.8–33.8)
85 years and older	19.3 (12.9–27.7)	19.0 (11.7–29.3)

When assessed in terms of affected teeth, the burden of carious disease was greatest in middle adulthood, particularly among males aged 40–44 years. In contrast, the lowest burden was observed in children aged 12 years for both sexes.

**Fig. 3:**
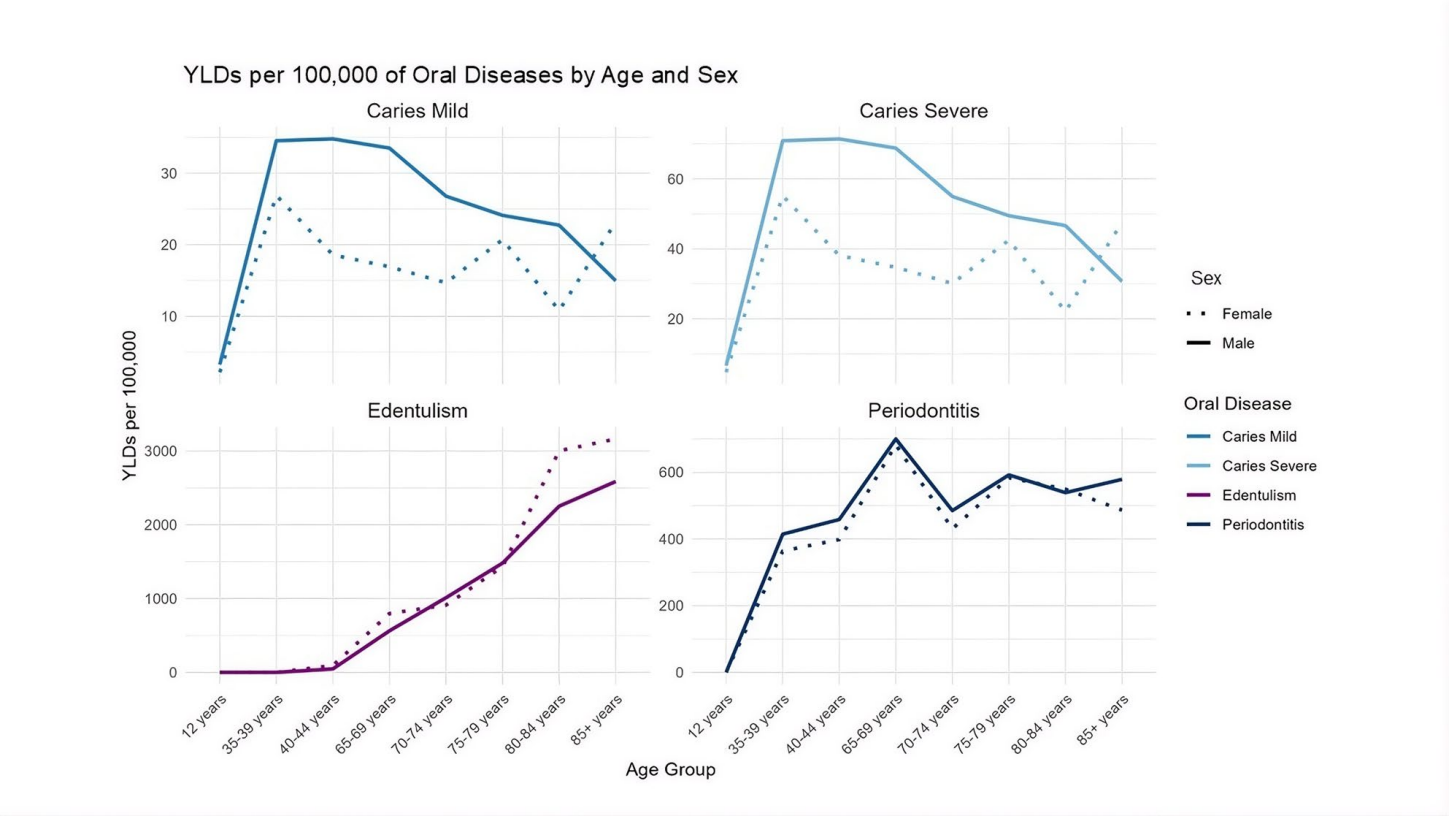
YLDs per 100,000 population across age groups, stratified by sex, for oral disease

The disability burden attributable to carious disease followed a similar age pattern. YLDs increased from childhood into adulthood, peaking in middle-aged adults before declining in older age groups (Fig. 3). Across all ages, severe carious disease contributed substantially more to disability than mild disease, often exceeding the latter by more than twofold. The highest burden was observed in the 40–44 age group, reaching 34.8 YLD per 100,000 for Mild Carious Disease and 71.4 YLDs per 100,00 for Severe Carious Disease. This indicates the exacerbating effects of both the acute and chronic phases.

Males consistently exhibit a higher burden of carious disease across all age groups than females, in both types of carious disease. Severe Carious Disease was seen to have a compounded, sometimes more than double, effect than mild carious disease for both sexes. The sex differences were minimal in childhood but becomes more pronounced in adults and older adults. The comprehensive breakdown and corresponding values are available in the Appendix (Table A1).

#### Periodontitis

The prevalence of periodontitis shows an age-related increase, hitting a peak in the 65–69 age group, with a prevalence of 100.0% in males and 97.5% in females (Table 2). The corresponding prevalence is nearly 2.4 million cases (2411642), and this progressively decreases with age – around 1.5 (1,532,533.23) million at 70–74 years, 735,438 at 80–84 years, and 522,066 among those aged 85 years and older.

The disability burden associated with periodontitis closely mirrored prevalence patterns, with the highest YLDs observed in older adults (Fig. 3). Males exhibited slightly higher YLDs than females across most age groups, although overall age trends were similar between sexes. Compared with carious disease, periodontitis accounted for a substantially larger proportion of total oral disease–related disability in adulthood and older age. Both sexes show a similar age trend with a peak in adulthood. The comprehensive breakdown and corresponding values are available in the Appendix (Table A2)*.*

**Table 2:** Prevalence of periodontitis (in %), with 95% confidence intervals (CI)

Age group	Female	Male
	% (95% CI)	% (95% CI)
12 years	-	-
35–39 years	52.0 (44.3–59.7)	59.2 (51.1–66.9)
40–44 years	56.9 (50.2–63.4)	65.5 (58.7–71.7)
65–69 years	97.5 (83.9–99.7)	100.0
70–74 years	61.5 (36.0–82.0)	69.3 (47.2–85.1)
75–79 years	83.4 (77.0-88.2)	84.6 (78.7–89.1)
80–84 years	78.5 (67.8–86.4)	77.0 (65.3–85.6)
85 years and older	69.5 (56.1–80.2)	82.7 (70.5–90.5)

#### Edentulism

The prevalence of edentulism shows an increase in prevalence with age. This is seen as the first measurable case of edentulism in the 40–44 age group with 18,278,008 cases in male and an almost double, 36,024 cases in females. There is a steady increase of 5–10% per age group with increase in age, across both sexes.

The highest prevalence is in the highest age group (85 years and older), more for females where nearly 47.2% of the population is seen to suffer from total tooth loss, resulting in an approximate 715,703 cases. The males by comparison show a 38.6% percent prevalence, as seen in Table 3, which amounts to roughly about 16,326,085 cases.

**Table 3:** Prevalence of edentulism (in %), with 95% confidence intervals (CI)

Age group	Female	Male
	% (95% CI)	% (95% CI)
12 years	-	-
35–39 years	-	-
40–44 years	1.4 (0.4–5.4)	0.7 (0.1–4.9)
65–69 years	11.9 (7.9–17.4)	8.4 (5.0-13.7)
70–74 years	13.6 (9.7–18.9)	15.1 (10.5–21.1)
75–79 years	21.4 (16.4–27.5)	22.1 (17.0-28.4)
80–84 years	44.8 (36.5–53.5)	33.6 (25.1–43.2)
85 years and older	47.2 (38.3–56.2)	38.6 (28.5–49.8)

Correspondingly, edentulism generated the highest YLD burden among all oral conditions in older age groups (Fig. 3). The disability associated with total tooth loss increased exponentially with age and exceeded that of both carious disease and periodontitis in the oldest populations. Females experienced nearly twice the burden of edentulism-related disability compared with males in advanced age. Both sexes show a steady increase in prevalence and burden with an increase in age (Fig. 3). The comprehensive breakdown and corresponding values are available in the Appendix (Table A3).

A brief analysis of the socio-economic status of individuals and prevalence of oral disease shows a correlation between low-income groups and higher prevalences. The figure is provided in the Supplementary Material (Figure A1). More males were seen to suffer from carious disease (24.5%) if they were from a low-income group as opposed to a high-income group (14.2%). This is also seen in periodontal disease and edentulism where there is a 75.5% and 19.5% prevalence in low-income groups and 61.6% and 4.7% prevalence in high- income groups, respectively. Females also show a similar predisposition of the low-income groups to have higher prevalence of oral disease as compared to high-income groups. 3.7% of high-income females are seen to suffer from edentulism.

**Fig. 4:**
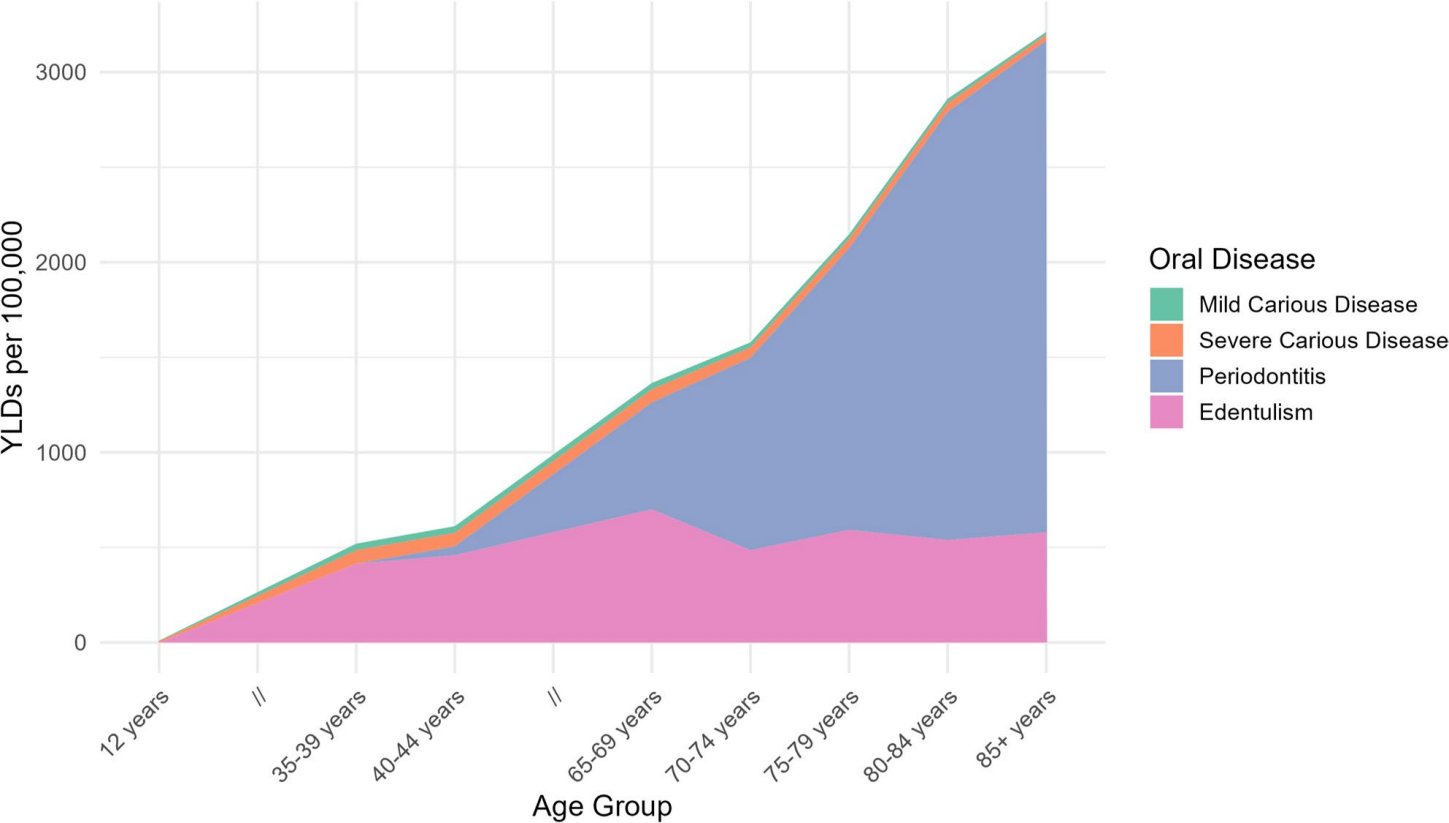
Overall burden of oral diseases as total YLDs of oral diseases per 100,000, stratified by age for males

**Fig. 5:**
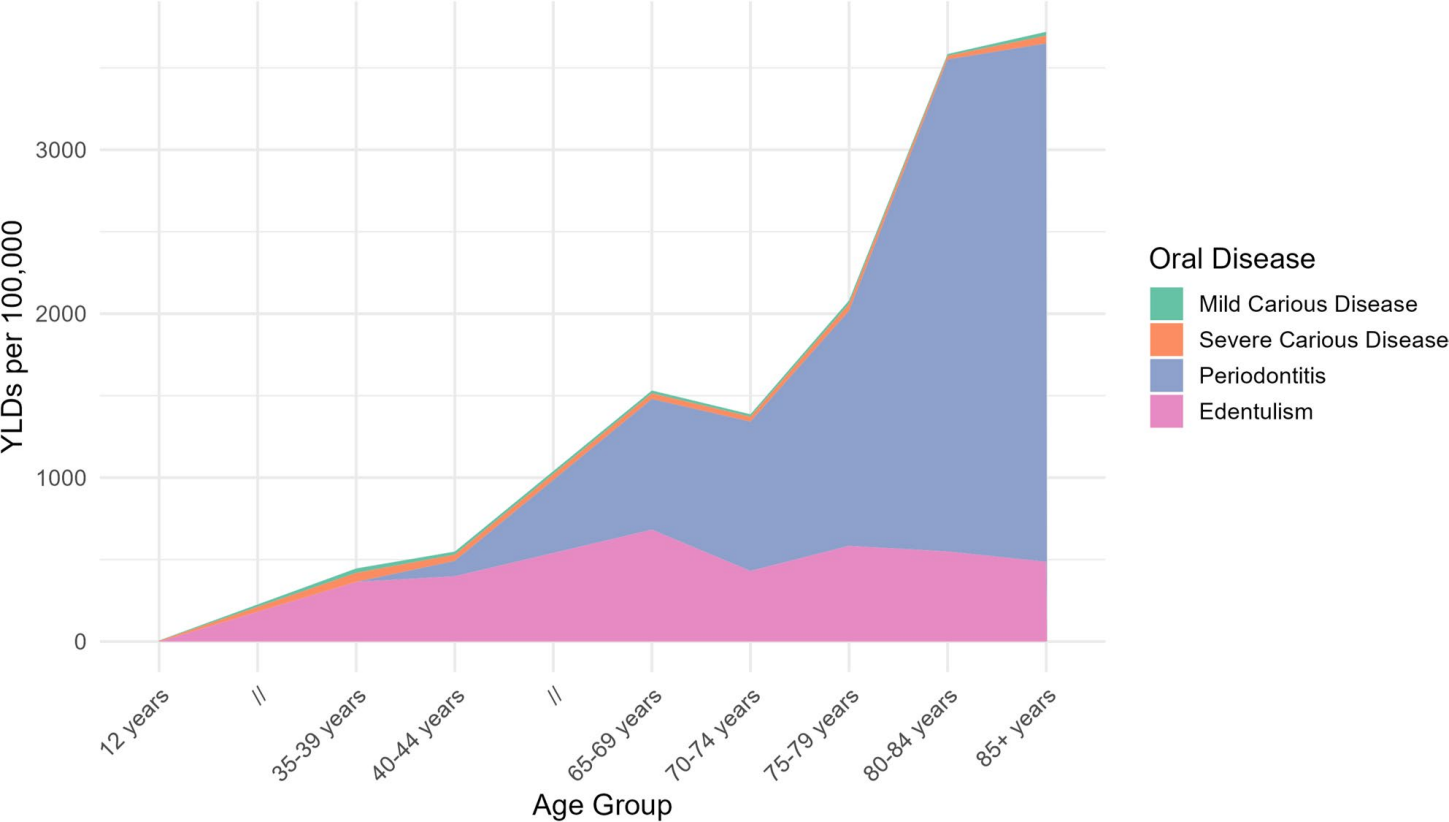
Overall burden of oral diseases as total YLDs of oral diseases per 100,000, stratified by age for females

Figures 4 and 5 display the overall burden of oral disease across age groups, for males and females, respectively. Males initially show a higher burden of oral disease but from approximately 45 years of age females suffer from an overall worser burden of oral disease throughout their lives. Mild and Severe Carious Disease are not as discernible since they comparatively exert lesser burden than Periodontitis and Edentulism (Fig. 4).

A comparison is also drawn between prevalence and YLD estimates in 2014 from GBD and the independent analyses using DMS V data, the figures which are included in the Supplementary Material (Figure A2-A7). The prevalence is seen to be higher for carious disease from the DMS dataset, with the corresponding YLDs per 100,000 also higher, especially severe carious disease. The prevalence and YLDs per 100,000 is also higher for the analysis of periodontitis from the DMS dataset as compared to GBD. The comparison for edentulism seems to not display strong differences.

## Discussion

This study highlights age-related trends among prevalence and burden of three major oral diseases in Germany. Carious disease was more prevalent among males aged 40–44 years, with the highest number of affected teeth and YLDs. Females displayed this peak at a younger age group of 35–39 years. Periodontitis shows an age increase, with a peak in prevalence and YLDs at 65–69 years for both sexes. Edentulism shows the strongest age gradient, with nearly half the females aged 85 years and older to be affected by the condition. There are also socio-economic disparities present with individuals from the lower income group who are seen to have a higher prevalence of disease.

Oral diseases affect individuals and populations across the entire life course [1, 7]. Prevalence of caries in permanent teeth typically shows steep increases after eruption and reaches the highest levels in late adolescence and early adulthood before remaining stable for the rest of the lifetime [5, 24]. Severe periodontal disease is a disease of middle age, reaching highest prevalence rates around 60 years. Edentulism (total tooth loss) steadily increases with a peak in older age groups [1, 25].

Toothache, which accompanies many of the major oral diseases, is a common individual experience and is consistently rated among the most intense of pains [1]. In most low- and middle-income countries, the reason for a dental visit often tends to be complaint oriented as opposed to behaviour oriented, which is evident from the degree of pain experienced due to toothache [1, 7]. Severe untreated oral diseases may negatively affect employment opportunities and reduce productivity [26]. There are also compounding physical, social and behavioural effects [6, 7]. All of these negative impacts of oral diseases disproportionally affect people from more disadvantaged backgrounds [1, 7].

The above findings highlight the burden of three main oral conditions, specifically carious disease, periodontitis and edentulism. DMFT at 12 years of age is recognized as the leading indicator of the oral health in children and adolescents [24]. There is a noticeable higher burden coming from periodontitis and edentulism, which also increases proportionally with an increase in age. A comparison from earlier research published by the IDZ which analyzed trends in carious experience shows a decline in prevalence of carious conditions since 1997 [27]. At the end of the 1980s, the introduction of individual and group prophylaxis for children and adolescents in Germany laid the foundation for a paradigm shift from reparative to preventative dental health care [5, 27].

Periodontitis prevalence estimates are highly sensitive to the clinical case definition employed. The broad threshold of an attachment loss greater than 4 mm captures cumulative tissue destruction and could explain the observed near-universal prevalence in older adults [28]. The periodontal status has also improved in comparison to the first DMS study for the younger age groups [19]. The age group of 65–74 displays the highest disability of 682.5 years for females and 700 years for males per 100,000. This is supported by the fact that this age group has a common predisposition to severe periodontal disease and early onset of tooth loss and compromised tooth attachment [19]. Age-related increases in periodontal attachment loss reflect cumulative lifetime exposure to risk factors, which can result in very high prevalence in older cohorts [29]. A study in Western Norway assessed and reported a 100% prevalence of periodontitis across a 70-year-old population [28]. 82.3% of these reported cases had a PPD of greater than 4 mm, which is the standard measurement also employed for this study [28].

This could imply a need for more focus on periodontal management concepts such as disease detection (screening) [19]. The consequence of ongoing periodontitis eventually leads to tooth loss, which is seen to exert a considerable burden, thus greatly reducing the quality of life [22]. This trend is present also globally, especially in association with low-income level and periodontitis needs to be considered as an intermediate factor leading to tooth loss [7, 30].

The prevalence of edentulism has halved since 1997, however it is still present in high number amongst women of the elderly age group [30, 31]. Complete tooth loss is seen as a phenomenon of an older population, especially in high-income countries and so the prevalence is expected to rise with an increase in age [31]. It has been shown to strongly affect masticatory function, quality of life and general health [22, 31]. Previous research has shown that each tooth that remains in the oral cavity after the age of 70 decreased the risk of mortality over 7 years by 4% [32]. The total state of tooth loss is considered as a ‘final target event’, because despite the closeness of gradual tooth loss and edentulism, there are epidemiological distinctions between these two states [31].

The burden of these three conditions has been observed to increase with age. For carious disease and periodontitis, males showed a higher prevalence and burden of disease, when associated with a low-income group, as supported by previous research [24].

On the other hand, females, especially of low socio-economic status showed a stronger predisposition to edentulism. This is supported by previous literature were being edentulous is associated with female sex in lower education levels and if they were a current or former smoker [7, 30]. Women have also been reported to experience a lower quality of care, as of 2017 [33]. Research has often also shown historical patterns of biological vulnerability and often limited access to dental care [30, 31, 34]. Further analysis can be recommended to assess YLD distribution based on socio-economic status to support public health reforms. Individuals with higher income experiencing lesser health outcomes is consistent with social determinants of health in previous literature and over the years [1, 7, 35].

The GBD study employs a Bayesian disease meta-regression algorithm (DisMod-MR 2.1) to obtain prevalence estimates. This is to account for the inconsistencies in data availability and heterogeneity of available data [2]. In the comparison between GBD and DMS data analysis, the differences in carious disease can be partially attributed to the type of analysis done. The burden assessed using DMS V focuses on number of teeth affected as opposed to the number of people affected. There is a higher estimate in this case because one person can have multiple carious teeth. The GBD reports number of people with the disease and does not differentiate between the number of carious lesions one person may have. For periodontitis, the GBD analysis only considers Class 4 from the scoring range of the CPITN i.e. presence of pockets 6 mm or deeper [12, 20, 21]. In this study, all individuals with pockets larger than 4 mm and above i.e. Class 3 and Class 4 are considered as diseased individuals. Thus, the differences among prevalence in DMS V and GBD analysis can be attributed to the severity level chosen for analysis.

GBD uses modelling to account for data inconsistencies and heterogeneity and also to make it comparable across countries and time points [12]. The DMS V data is nationally representative and so more specific to Germany oral health status.

The YLD estimate is stratified by severity of the condition in case of carious disease and also considers the weight of filled teeth. This refines the distribution of the burden which is not present in GBD. The severity stratification in GBD is fixed and globally averaged [12].

Oral health has been closely associated with general health, as they share common pathways [1]. There is evidence of associations between periodontal disease and cardiovascular conditions, diabetes mellitus and often certain cancer conditions [1, 36–38]. The comparison between burden exerted by Oral Diseases and other diseases, which include various types of cancers, mental health conditions, road traffic accidents and other noncommunicable conditions, elucidates the importance of oral health [4]. Periodontitis and Edentulism contribute significantly to this burden, and as a result the impact is seen in the older age groups. A report as of 2015 found that oral conditions accounted for more health loss than 35 of 39 categories of cancer [39]. Suboptimal oral health, including functional problems have consequences to quality of life [40].

Drawing from the GBD study of 2019, a similar analysis was conducted in Brazil to also assess the prevalence and disability caused by oral diseases [41]. The analysis was similarly done from modelling strategy employed by GBD to obtain the estimates. All oral diseases combined ranked eighth among all causes of disability, causing more than 970,000 YLDs – out of which edentulism alone caused more than 600,000 YLDs, followed by periodontitis, with almost 194,000 YLDs. Untreated caries in permanent teeth caused more than 51,000 YLDs and approximately 5,000 YLDs in primary teeth. Nearly 100 million Brazilians were found to have least one event regarding any oral disorder assessed in 2019, which was equivalent to 45.26% of the population at the time [41]. Our findings are consistent with this evidence, highlighting the marked impact oral diseases can have on an individual’s quality of life.

There are both strengths and limitations present in this study. The common difficulty of the globally used index for recording caries experience (DMFT index) in epidemiological studies is the differentiation between actual causes of tooth loss i.e. if a tooth has been actually lost to carious disease or some other condition. The M component of the index often overestimates the carious experience [5]. In this study, this is compensated by using the T-Health index for assessing the prevalence and burden.

A major strength of this study is the use of data from the Fifth German Oral Health Study (DMS V), a nationally representative epidemiological survey based on standardized clinical examinations rather than exclusively modelled estimates. This allows for a robust, ground-truthed assessment of the prevalence and burden of major oral diseases across children as well as multiple adult and older age groups in Germany. Stratification based on socio-economic status also provides insights into oral health inequalities which can be of public health relevance.

The comparative analysis between burden estimates derived from DMS V and those reported by the GBD study strengthens the international relevance of the results. This comparison illustrates how nationally collected clinical data can complement global modelling approaches by validating estimates, identifying potential underestimation of disease burden, and improving contextual interpretation in high-income settings. The dataset also contains information on oral mucosal lesions, dental erosions and molar incisor hypomineralization (MIH) which may exert burden on general health and quality of life [42]. The recent DMS 6 dataset also has an orthodontic focus which provides valuable information as dento-facial malocclusion which is known to impact quality of life [5, 43]. With the inclusion of other age groups and variables, the generalisability of the dataset and its usefulness for burden of disease calculations would be improved as it would encompass dental health and condition over the whole age span.

DMS V data are cross-sectional in nature, which precludes causal inference and limits the ability to examine disease progression or changes over time within the same individuals. Consequently, observed age-related patterns should be interpreted as differences between age cohorts rather than true longitudinal trends.

The conducted analysis is restricted to the age groups included in DMS V. As a result, the generalizability of the findings is limited, and the burden of oral diseases in the missing cohorts are not covered by this analysis. While these gaps are addressed in the subsequent DMS 6 study, the present results do not capture the full life-course burden of oral disease in Germany.

The limitation caused by the DMFT index as measured above is partly mitigated by using the T-Health Index. However, for periodontitis, DMS V study evaluated treatment needs for periodontitis under the perspectives of German healthcare solely based on PD/PPDs [3]. PPD does not always necessarily reflect clinical attachment loss; in case of periodontal overgrowth, it often overestimates the actual loss. In the elderly, by contrast, PPD plateaus and thus could underestimate the burden [20]. In many settings, clinical attachment loss rather than PPD may be used to decide on treatment needs and resulting demands, and even in Germany, periodontal therapy may be provided to teeth not falling into our case definition outside of the statutory insurance setting (i.e., patients would pay privately for such therapy) [20]. Also, the proportion of treated cases remains unclear [19]. For example, in a treated case, a PPD of 4 mm may reflect an acceptable endpoint of treatment which does not generate further treatment need [19]. Dichotomous Periodontal Index or Genetic Susceptibility Index for Periodontal needs (GSI) could be two alternatives to consider for a more accurate assessment [20]. In the present analysis, people with loss of only all teeth have been considered to be edentulous. Individuals with varying levels of partial edentulism or fewer than minimum number of functional teeth required for adequate oral function are overlooked. Previous research has shown that tooth numbers below a minimum of 20 teeth, with 9 to 10 pairs of contacting units, are associated with impaired masticatory efficiency, performance, and masticatory ability (an individual’s perception of his/her ability to chew) [34].

With the information present regarding socio-economic status, only the prevalence in terms of percentage by age and sex was analyzed. Further research can be done to quantify the burden based on income groups, which can yield useful information for public health measures. National epidemiological data such as those from DMS V and the more recent DMS 6 offer clinically detailed, population-representative measurements, which when incorporated with GBD estimates can reveal useful information regarding the health status of a population.

In contrast to the DMS epidemiological study, most dental practitioners in German clinical practice determine the periodontal stage based on radiographs and not on Clinical Attachment Loss (CAL). In radiographs, only a significant amount of bone destruction can be detected (the difference ranging from 0 to 1.6 mm) [44]. The oral health situation in Germany has improved significantly over the past two decades with a notable decline in the number of edentate individuals and an increase in the average number of teeth [45]. More participants in later waves were highly educated, which may have facilitated a more comprehensive understanding of the contents of periodontal treatment. The level of knowledge of the German population regarding periodontal health and treatment used to be low. Higher rates of tooth loss observed during periodontal treatment may be attributable to inadequate oral hygiene instruction or the inefficacy of nonsurgical periodontal treatment. In addition, less effort was often made to retain teeth with poor prognosis during active periodontal therapy in the earlier waves [46]. Supportive Periodontal Care (SPC) is also a structured maintenance program which was covered by statutory insurance in Germany only as of July 2021. This could also be a contributing factor in willingness of patients to seek specific periodontal treatment [46].

Similar to other high-income countries, Germany has experienced notable shifts in the prevalence of upstream health determinants over the past decades: the proportion of individuals with higher education has increased, the prevalence of tobacco consumption has declined, and the prevalence of diabetes has increased [46]. There has been an increase in utilization of toothbrushes and interdental cleaning aids. The decrease in orally diseased individuals from the earlier DMS studies to the most recent DMS VI study could be attributed to an increased educational status, the proportion of non-smokers and increased usage of dental cleaning products. Population wide preventive measures such as smoking bans have provided greatly successful in this case [46].

One third of those requiring care in Germany are very old [47]. Geriatric specific dental care is therefore also a section that requires more targeted measures and initiatives. In terms of treatment, there has been a positive shift from more patients preferring fixed denture prosthesis (with or without the support of implants) rather than removable denture prosthesis. This shift is also supported with a higher education status [48]. As more teeth are retained into old age, the challenges for dental care increase, which include managing periodontal disease, root caries, and prosthodontic restoration. In the future, equal-opportunity, accessible access to dental care must be provided for the heterogenous group of seniors, particularly in undersupplied and rural areas [47]. The health care system, especially at the interface of medical outpatient and inpatient care, must offer not only dental treatment but also oral care to achieve optimal oral health for people in challenging life circumstances [47].

## Conclusions

This study gives a comprehensive overview of the burden of oral diseases in Germany, with distinct patterns across carious disease, periodontitis, and edentulism. While carious disease remains prevalent across the life course, its burden peaks in mid-adulthood and declines in older age groups. In contrast, periodontitis and edentulism contribute disproportionately to disability in older adults, particularly among those aged 65 years and above, highlighting the growing impact of these conditions in an ageing population.

There were sex predilections, with males generally exhibiting a higher burden of carious disease and periodontitis. Females experienced a greater prevalence and burden of edentulism, especially in advanced age. Additionally, individuals from lower income groups consistently showed higher prevalences of all three conditions, underscoring persistent socio-economic inequalities in oral health.

These findings emphasize the need for a stronger public health focus on periodontal disease prevention, early detection, and long-term management, particularly among older adults. Decreases in the rates of oral conditions need to surpass rates of population growth and ageing to stabilize or even decrease the burden on population health and the health systems [2]. The continual assessment from 1997 shows the positive effect of prevention-based approaches in carious disease, however this needs to be improved more in cases of periodontitis and total tooth loss, especially for older age groups [5, 16]. Future research can be focused on filling the existing data gaps and analyzing burden with ongoing treatment, other oral conditions and demographic variables in order to better implement specific dental public health aims.

## Supplementary Information

Supplementary Material 1.

## Data Availability

The authors state that access restrictions apply to the DMS V data from the Institute of German Dentists on which the results are based. Due to strict data protection regulations, DMS V data are generally not publicly available. Therefore, the minimal dataset underlying the results of this study is archived but cannot be made available to other researchers.

## Abbreviations

CPI: Community Periodontal Index
CPITN: Community Periodontal Index of Treatment Needs
CAL: Clinical Attachment Loss
DALY: Disability-Adjusted Life Years
DMFT: Decayed, Missing and Filled Teeth
DMS: Deutsche Mundgesundheitsstudie
DT: Decayed Teeth
DW: Disability Weights
FT: Filled Teeth
GBD: Global Burden of Disease
IDZ: Institute of German Dentists (Institut der Deutschen Zahnärzte)
NCD: Noncommunicable Disease
PD: Pocket Depth
PPD: Periodontal Pocked Depth
SPC: Supportive Periodontal Care
WHO: World Health Organization
YLD: Years Lived with Disability
