# Supplementary material for: Assessing the burden of oral health conditions in the German population: a focus on dental caries, periodontitis, and edentulism
Source: BMC Oral Health. 2026 Mar 19;26:614. doi: 10.1186/s12903-026-08134-8 (PMC13064231; doi:10.1186/s12903-026-08134-8)
Supplement: Supplementary file 1 — Supplementary Material 1. [file 12903_2026_8134_MOESM1_ESM.docx]

Supplementary Materials

**Appendix A1. List of sources used by GBD, Accessed 16^th^ February 2025**

| Citation |
| --- |
| Holtfreter B, Demmer RT, Bernhardt O, Papapanou PN, Schwahn C, Kocher T, Desvarieux M. A comparison of periodontal status in the two regional, population-based studies of SHIP and INVEST. J Clin Periodontol. 2012; 39(12): 1115-24. |
| Hohlfeld M, Bernimoulin JP. Application of the community periodontal index of treatment needs (CPITN) in a group of 45-54-year-old German factory workers. J Clin Periodontol. 1993; 20(8): 551-6. |
| Gaengler P, Goebel G, Kurbad A, Kosa W. Assessment of periodontal disease and dental caries in a population survey using the CPITN, GPM/T and DMF/T indices. Community Dent Oral Epidemiol. 1988; 16(4): 236-9. |
| Wagner Y, Heinrich-Weltzien R. Caries Prevalence and Risk Assessment in Thuringian Infants, Germany. Oral Health Prev Dent. 2017; 15(5): 489-494. |
| Zerfowski M, Koch MJ, Niekusch U, Staehle HJ. Caries prevalence and treatment needs of 7- to 10-year-old schoolchildren in southwestern Germany. Community Dent Oral Epidemiol. 1997; 25(5): 348-51. |
| Sönju Clasen AB, von der Fehr FR, Kant van Daal JM. Caries prevalence of kindergarten children in Salzgitter and Oslo. Caries Res. 1992; 26(3): 201-4. |
| Schutzhold S, Kocher T, Biffar R, Hoffmann T, Schmidt CO, Micheelis W, Jordan R, Holtfreter B. Changes in prevalence of periodontitis in two German population-based studies. J Clin Periodontol. 2015; 42(2): 121-30. |
| Bolin AK, Bolin A, Koch G. Children's dental health in Europe: caries experience of 5- and 12-year-old children from eight EU countries. Int J Paediatr Dent. 1996; 6(3): 155-62. |
| Grund K, Goddon I, Schuler IM, Lehmann T, Heinrich-Weltzien R. Clinical consequences of untreated dental caries in German 5- and 8-year-olds. BMC Oral Health. 2015; 15(1): 140. |
| Flores-de-Jacoby L, Mengel R, Joannou U, Zafiropoulos GG. CPITN application in regular dental practice. Dtsch Zahn Mund Kieferheilkd Zentralbl. 1992; 80(1): 13-20. |
| Robke FJ. Effects of nursing bottle misuse on oral health. Prevalence of caries, tooth malalignments and malocclusions in North-German preschool children. J Orofac Orthop. 2008; 69(1): 5-19. |
| Weusmann J, Mahmoodi B, Azaripour A, Kordsmeyer K, Walter C, Willershausen B. Epidemiological investigation of caries prevalence in first grade school children in Rhineland-Palatinate, Germany. Head Face Med. 2015; 11: 33. |
| Holtfreter B, Schwahn C, Biffar R, Kocher T. Epidemiology of periodontal diseases in the Study of Health in Pomerania. J Clin Periodontol. 2009; 36(2): 114-23. |
| Bissar A, Schiller P, Wolff A, Niekusch U, Schulte AG. Factors contributing to severe early childhood caries in south-west Germany. Clin Oral Investig. 2014; 18(5): 1411-8. |
| European Commission (2012): Eurobarometer 59.0 (Jan-Feb 2003). European Opinion Research Group (EORG), Brussels. GESIS Data Archive, Cologne. ZA3903 Data file Version 1.0.1, doi:10.4232/1.11352 |
| World Health Organization (WHO). Germany World Health Survey 2004. Geneva, Switzerland: World Health Organization (WHO), 2005. |
| Pitchika V, Kokel C, Andreeva J, Crispin A, Hickel R, Garcia-Godoy F, Kuhnisch J, Heinrich-Weltzien R. Longitudinal study of caries progression in 2- and 3-year-old German children. Community Dent Oral Epidemiol. 2016; 44(4): 354-63. |
| Guarnizo-Herreño CC, Tsakos G, Sheiham A, Watt RG. Oral health and welfare state regimes: a cross-national analysis of European countries. Eur J Oral Sci. 2013; 121(3 Pt 1): 169–75. |
| Borutta A, Brauner K, Hufnagl S, Márton S, Mavrodisz K, Tarján I. Oral health in 8-9 year-old children in Saxony-Anhalt (Germany) and in two Hungarian cities (Budapest and Debrecen). Community Dent Health. 2006; 23(1): 26-30. |
| Schiffner U, Hoffmann T, Kerschbaum T, Micheelis W. Oral health in German children, adolescents, adults and senior citizens in 2005. Community Dent Health. 2009; 26(1): 18-22. |
| Micheelis W, Bauch J. Oral health of representative samples of Germans examined in 1989 and 1992. Community Dent Oral Epidemiol. 1996; 24(1): 62-7. |
| Flores-de-Jacoby L, Schoop S, Weichsler C, Zafiropoulos GG. Periodontal conditions in Hesse, Federal Republic of Germany, measured by CPITN. Community Dent Oral Epidemiol. 1989; 17(6): 307-9. |
| Mengel R, Koch H, Pfeifer C, Florès-de-Jacoby L. Periodontal health of the population in eastern Germany (former GDR). J Clin Periodontol. 1993; 20(10): 752-5. |
| Holtfreter B, Kocher T, Hoffmann T, Desvarieux M, Micheelis W. Prevalence of periodontal disease and treatment demands based on a German dental survey (DMS IV). J Clin Periodontol. 2010; 37(3): 211-9. |
| Pieper K, Dressler S, Heinzel-Gutenbrunner M, Neuhäuser A, Krecker M, Wunderlich K, Jablonski-Momeni A. The influence of social status on pre-school children's eating habits, caries experience and caries prevention behaviour. Int J Public Health. 2012; 57(1): 207-15. |

**Table A1. Prevalence and YLDs per 100,000 of Carious Disease with 95% CI**

| Age Group |  | Male |  |  |  | Female |  |  |
| --- | --- | --- | --- | --- | --- | --- | --- | --- |
|  | Prevalence of Carious Disease *(in number of teeth)* | Prevalence of Carious Disease per 100,000 *(in number of teeth)* | YLDs per 100,000 of Carious Disease Mild | YLDs per 100,000 of Carious Disease Severe | Prevalence of Carious Disease *(in number of teeth)* | Prevalence of Carious Disease per 100,000 *(in number of teeth)* | YLDs per 100,000 of Carious Disease Mild | YLDs per 100,000 of Carious Disease Severe |
| 12 years | 76386.57917  [60923.809634072, 91849.34871196] | 20292.26505  [16184.54585,24399.98425] | 3.483481656  [2.77832802044378,4.18863529239625] | 7.152822468  [5.70489213002131,8.60075280521195] | 48403.85104  [36655.909426899,60151.800143232] | 13572.3021  [10278.2127,16866.3936] | 2.174911435  [1.76441443641071,2.89537774967678] | 4.784102078  [3.62296818745242,5.94523670928226] |
| 35-39 years | 5861588.815 [3622171.359, 8865437.381] | 200993.7515  [124204.1762, 303995.6528] | 34.52756017  [21.32157097, 52.18556319] | 70.85609532  [43.78074207, 107.155457] | 4484355.531  [2909794.381, 6608697.981] | 156417.675  [101495.8044, 230516.3286] | 26.87009245  [17.42332716, 39.57169888] | 55.1417425  [35.7762659, 81.25472294] |
| 40-44 years | 5286713.769  [3690570.255, 7302331.055] | 202467.3388  [141339.2082, 279660.22] | 34.78069925  [24.26306466, 48.00800916] | 71.37557735  [49.82067115, 98.57745796] | 2781815.507  [1781324.01, 4206343.061] | 108109.9712  [69227.7712, 163471.5264] | 18.57158995  [11.88401938, 28.06241995] | 38.11188341  [24.40210376, 57.62209413] |
| 65-69 years | 4701986.125 [3226679.038, 6586961.035] | 194970.32  [133795.9381, 273131.793] | 33.49282953  [22.96814549, 46.88730353] | 68.73266196  [47.16174314, 96.2762521] | 2562527.8  [1535776.461, 4107203.468] | 98491.03  [59027.7325, 157860.8045] | 16.91920738  [10.13302472, 27.09925262] | 34.72092917  [20.80669113, 55.64437023] |
| 70-74 years | 3211247.148  [1790313.691, 5217066.309] | 155896.3788  [86914.3384, 253272.8588] | 26.78054198  [14.92019263, 43.47820983] | 54.95797055  [30.63644354, 89.27617446] | 2022365.087  [1267381.483, 3117129.719] | 85463.3367  [53558.406, 131727.1093] | 14.6812549  [9.194130098, 22.61299897] | 30.12829148  [18.8788077, 46.43250146] |
| 75-79 years | 2541207.965  [1608947.556, 3803161.593] | 140281.5872  [88818.2785, 209944.8576] | 24.09816676  [15.24703345, 36.0402872] | 49.45330607  [31.30756357, 74.00348314] | 2770155.584  [1745830.22, 4189366.274] | 120556.8624  [75978.33665, 182320.7535] | 20.70976978  [13.04285852, 31.29818179] | 42.49977161  [26.78161122, 64.26626001] |
| 80-84 years | 1263815.086  [662036.1688, 2183844.627] | 132320.8629  [69314.8848, 228647.536] | 22.73063974  [11.89897378, 39.25089168] | 46.64692113  [24.43281044, 80.59599205] | 907474.9262  [410447.3698, 1791797.819] | 62949.6726  [28471.8914, 124293.3362] | 10.81376208  [4.887641235, 21.33687667] | 22.19157545  [10.03605975, 43.81217008] |
| 85+ years | 549823.9726  [254533.8891, 1058350.41] | 87097.102  [40320.47565, 167652.3] | 14.96191005  [6.921634276, 28.7801145] | 30.70424088  [14.21256836, 59.0957754] | 2043565.342 [953093.4334, 3819420.519] | 134771.4658  [62855.73375, 251887.6648] | 23.1516147  [10.79016044, 43.24041981] | 47.51082933  [22.15602366, 88.78790728] |

**Table A2. Prevalence and YLDs per 100,000 of Periodontitis with 95% CI**

| Age Group | Male |  | Female |  |
| --- | --- | --- | --- | --- |
|  | Prevalence of Periodontitis *(in number of cases)* | YLDs per 100,000 of Periodontitis | Prevalence of Periodontitis *(in number of cases)* | YLDs per 100,000 of Periodontitis |
| 12 years | - | - | - | - |
| 35-39 years | 1726451.968  [1490231.344,1951007.376] | 414.4  [357.7,468.3] | 1490793.72 [1270041.573,1711545.867] | 364  [310.1,417.9] |
| 40-44 years | 1710299.32  [1532741.528,1872190.248] | 458.5  [410.9,501.9] | 1464113.815 [1291713.77,1631367.59] | 398.3  [351.4,443.8] |
| 65-69 years | 2411642 | 700 | 2536743.3 [2182900.132,2593982.636] | 682.5  [587.3,697.9] |
| 70-74 years | 1427482.98  [972253.92,1752940.86] | 485.1 [330.4,595.7] | 1455307.71 [851887.44,1940410.28] | 430.5  [252,574] |
| 75-79 years | 1532533.23  [1425654.435,1614050.955] | 592.2 [550.9,623.7] | 1916365.2  [1769306,2026659.6] | 583.8  [539,617.4] |
| 80-84 years | 735437.78  [623689.442,817577.584] | 539  [457.1,599.2] | 1131646.58 [977396.664,1245532.032] | 549.5  [474.6,604.8] |
| 85+ years | 522066.079 [445050.285,571305.685] | 578.9  [493.5,633.5] | 1053841.705 [850654.959,1216087.838] | 486.5  [392.7,561.4] |

**Table A3. Prevalence and YLDs per 100,000 of Edentulism with 95% CI**

| Age Group | Male |  | Female |  |
| --- | --- | --- | --- | --- |
|  | Prevalence of Edentulism *(in number of cases)* | YLDs per 100,000 of Edentulism | Prevalence of Edentulism *(in number of cases)* | YLDs per 100,000 of Edentulism |
| 12 years | - | - | - | - |
| 35-39 years | - | - | - | - |
| 40-44 years | 18278.008  [2611.144,127946.056] | 46.9  [6.7, 328.3] | 36023.89 [10292.54,138949.29] | 93.8  [26.8, 361.8] |
| 65-69 years | 202577.928  [120582.1,330394.954] | 562.8  [335, 917.9] | 309612.772  [205541.252,452711.112] | 797.3  [529.3, 1165.8] |
| 70-74 years | 311038.86  [216285.3,434630.46] | 1011.7  [703.5,1413.7] | 321824.144 [229536.338,683876.306] | 911.2  [649.9, 1936.3] |
| 75-79 years | 400342.605  [307955.85,514467.42] | 1480.7  [1139, 1902.8] | 491729.2  [376839.2,631895] | 1433.8  [1098.8, 1842.5] |
| 80-84 years | 320918.304  [239733.614,412609.248] | 2251.2  [1681.7, 2894.4] | 645831.424  [526179.62,771249.58] | 3001.6  [2445.5, 3584.5] |
| 85+ years | 243672.922  [179913.945,314375.946] | 2586.2  [1909.5, 3336.6] | 715702.568  [580750.177,852171.278] | 3162.4  [2566.1, 3765.4] |

**Figure A1. Prevalence of Oral Diseases by Sex and Socio-economic status**


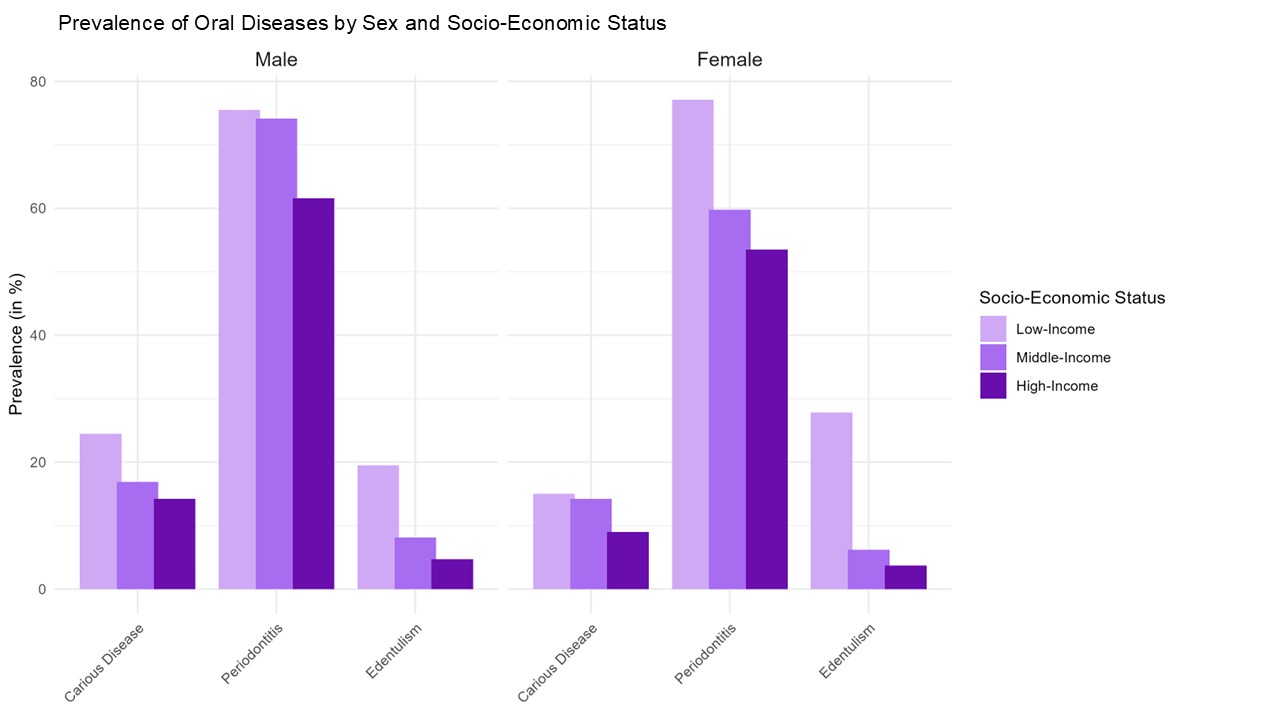


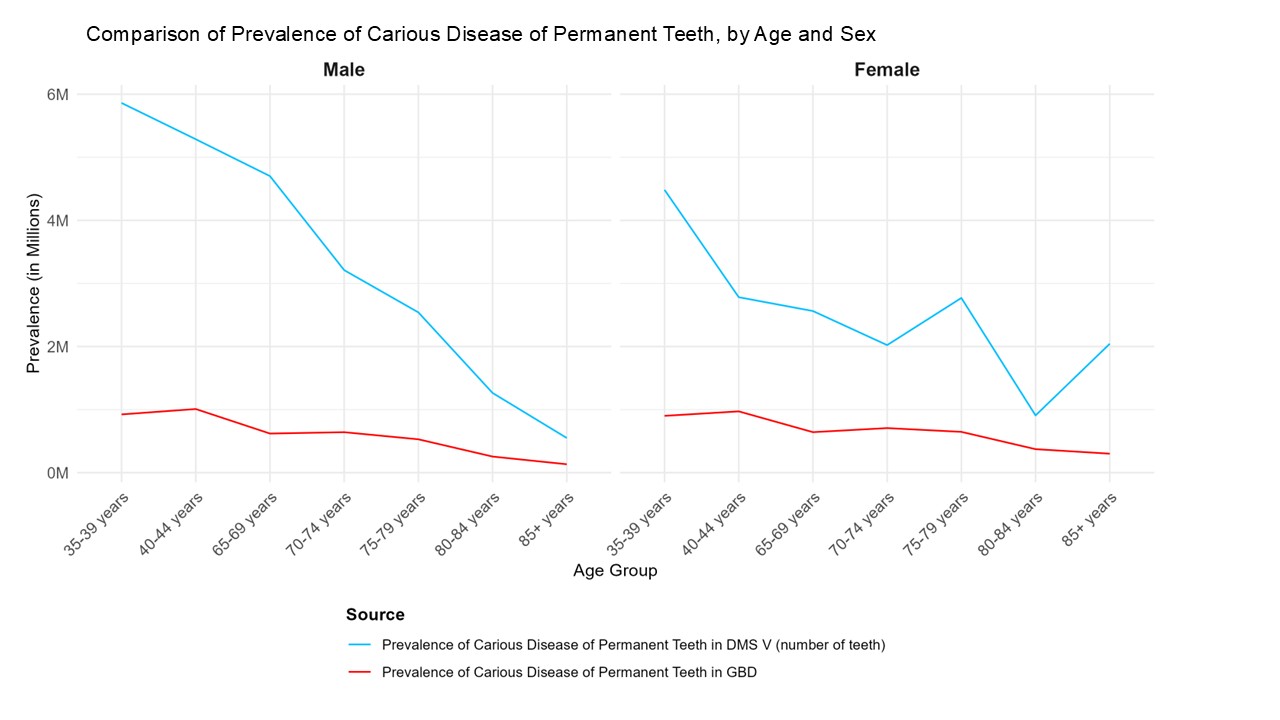
**Figure A2. Comparison of Prevalence of Carious Disease between GBD and DMS V. The age group of 12 has been omitted as GBD reports other age ranges for childhood and DMS V data has only 12 years of age; an appropriate comparison as a result cannot be drawn.**

**Figure A3. Comparison of YLDs of Carious Disease between GBD and DMS V. The age group of 12 has been omitted as GBD reports other age ranges for childhood and DMS V data has only 12 years of age; an appropriate comparison as a result cannot be drawn. The mild and severe carious disease has been displayed from the results in DMS V.**

**
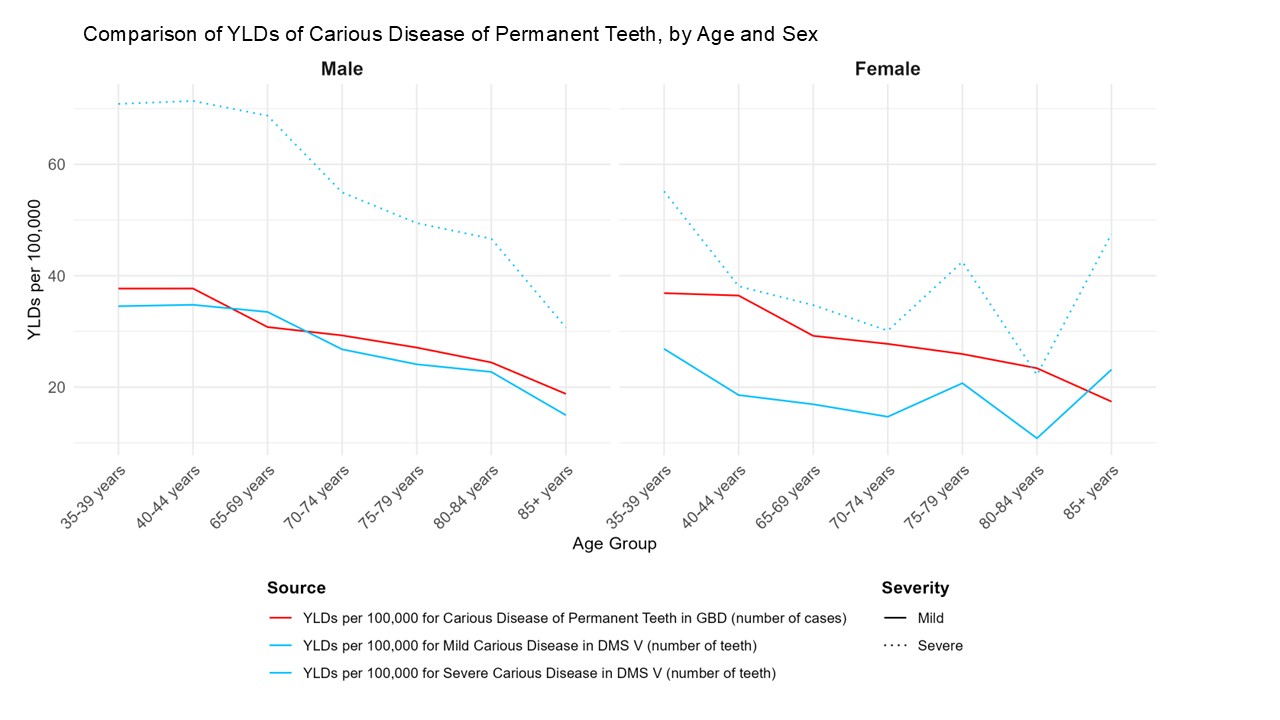
**

**Figure A4. Comparison of Prevalence of Periodontitis between GBD and DMS V.**


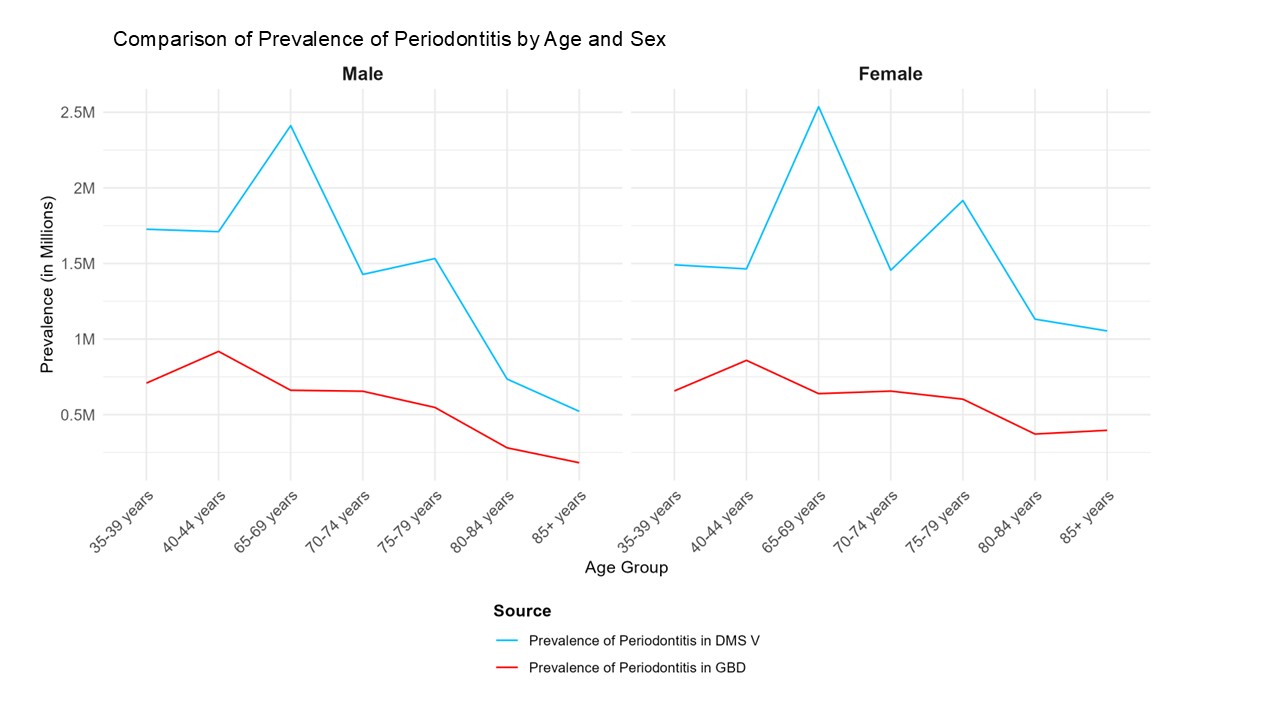


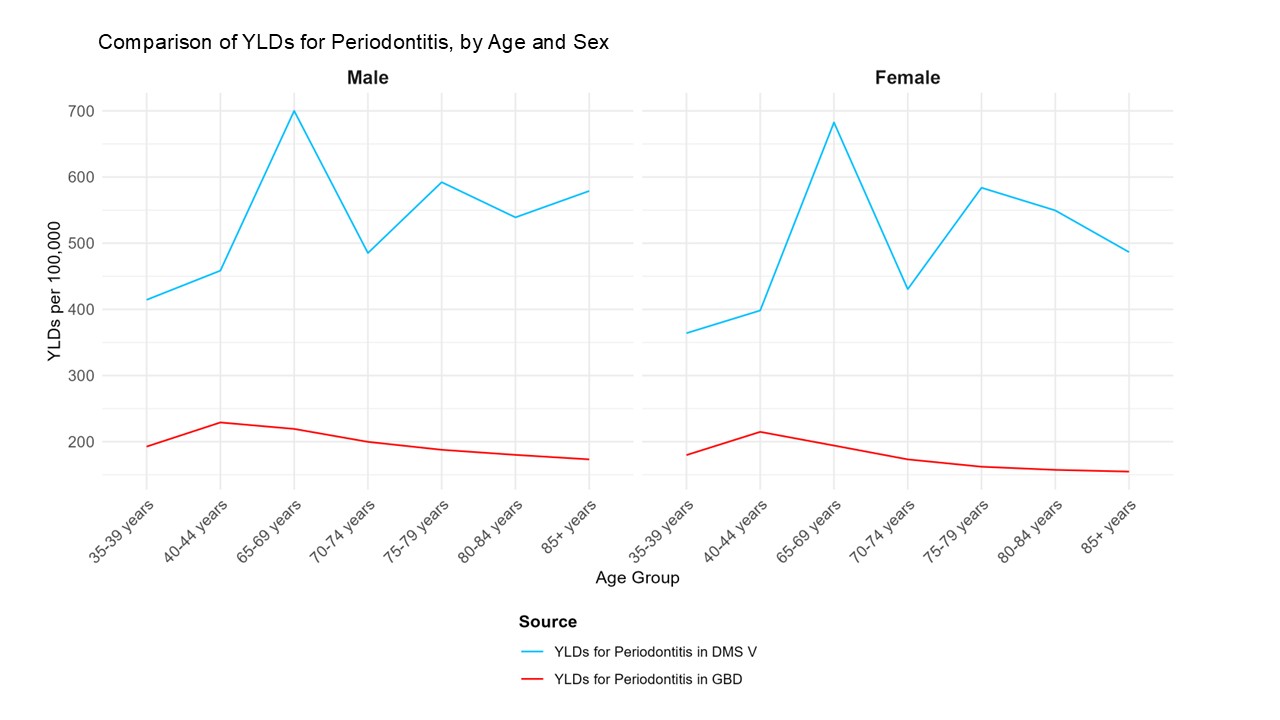
**Figure A5. Comparison of YLDs of Periodontitis between GBD and DMS V.**

**Figure A6. Comparison of Prevalence of Edentulism between GBD and DMS V.**


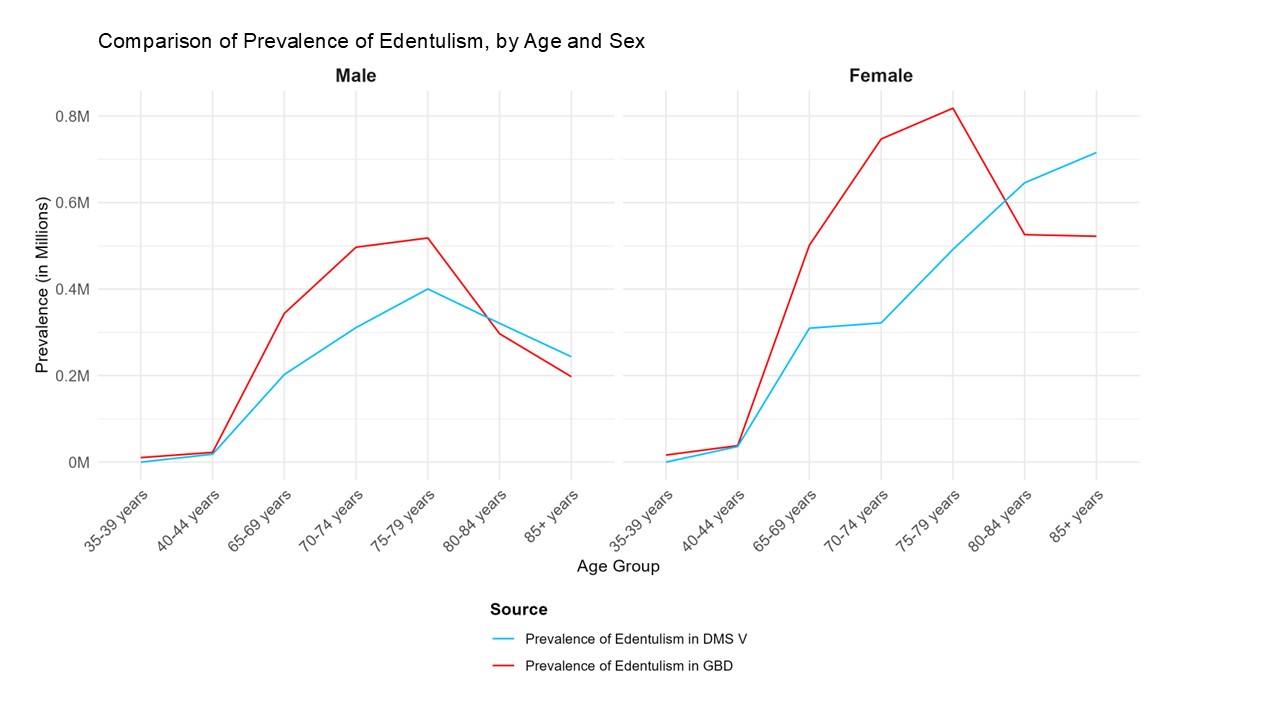


**
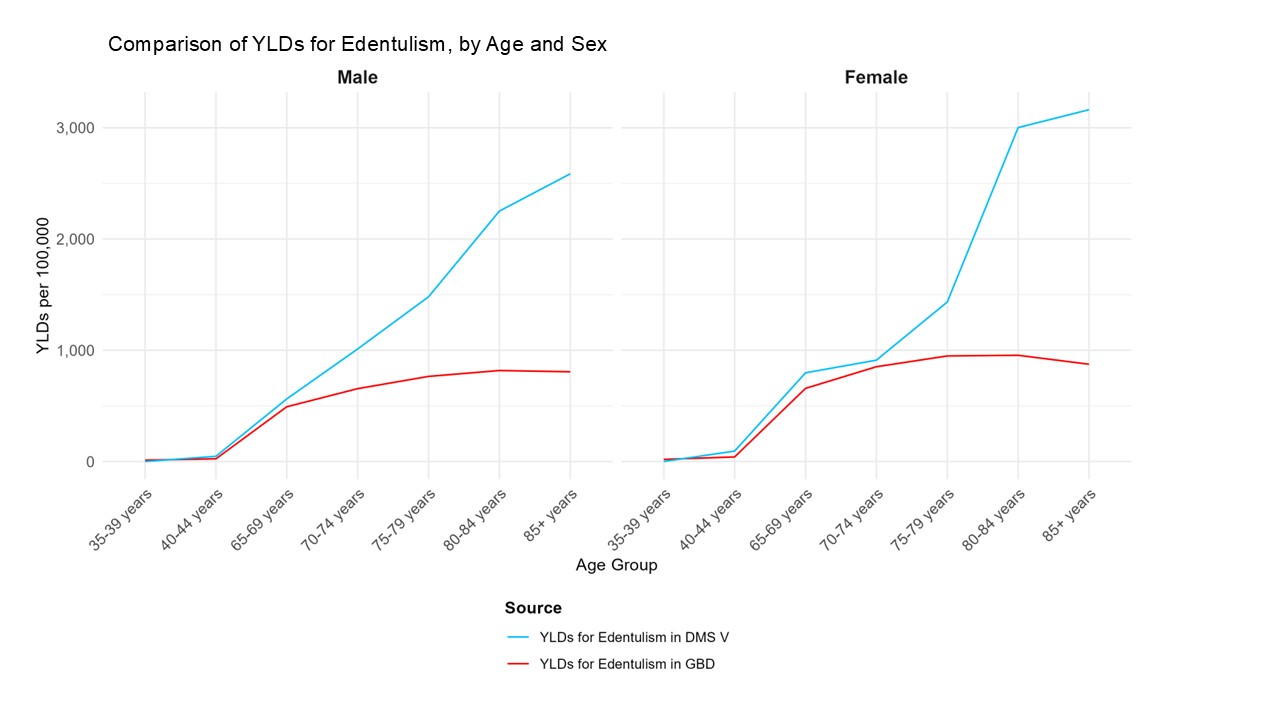
Figure A7. Comparison of YLDs of Edentulism between GBD and DMS V.**
